# Angular Rate Optimal Design for the Rotary Strapdown Inertial Navigation System

**DOI:** 10.3390/s140407156

**Published:** 2014-04-22

**Authors:** Fei Yu, Qian Sun

**Affiliations:** 1 Collage of Automation, Harbin Engineering University, 145 Nantong Avenue, Harbin 150001, China; E-Mail: yufei@hrbeu.edu.cn; 2 Department of Earth and Space Science and Engineering, York University, 4700 Keele Street, Toronto ON M3J 1P3, Canada

**Keywords:** rotary strapdown inertial navigation system, rotation modulation, rotating angular rate, optimal design

## Abstract

Due to the characteristics of high precision for a long duration, the rotary strapdown inertial navigation system (RSINS) has been widely used in submarines and surface ships. Nowadays, the core technology, the rotating scheme, has been studied by numerous researchers. It is well known that as one of the key technologies, the rotating angular rate seriously influences the effectiveness of the error modulating. In order to design the optimal rotating angular rate of the RSINS, the relationship between the rotating angular rate and the velocity error of the RSINS was analyzed in detail based on the Laplace transform and the inverse Laplace transform in this paper. The analysis results showed that the velocity error of the RSINS depends on not only the sensor error, but also the rotating angular rate. In order to minimize the velocity error, the rotating angular rate of the RSINS should match the sensor error. One optimal design method for the rotating rate of the RSINS was also proposed in this paper. Simulation and experimental results verified the validity and superiority of this optimal design method for the rotating rate of the RSINS.

## 1. Introduction

The operation of an inertial navigation system (INS) depends on Newton' second law [1]. The inertial navigation is the process whereby the measurements of inertial sensors (gyroscopes and accelerometers) calculate the position and velocity of the vehicle. Unlike other types of navigation systems, the INS is an entirely independent navigation system, since it is not dependent on the transmission of signals from the vehicle or reception from an external source. However, the fatal flaw of the INS is that the navigation error caused by the bias of the inertial sensors increases with time. Therefore, the precision of the INS is subject to the precision of inertial sensors [2,3].

For the original INS, the inertial sensors are mounted on a stable platform, which is mechanically isolated from the rotational motion of the vehicle [4]. Due to the mechanical complexity of the platform system, a new type of INS, which is called the strapdown inertial navigation system (SINS), is replacing the traditional platform inertial navigation system. The SINS has the advantages of lower cost, smaller size and greater reliability compared with platform systems, since the inertial sensors are attached rigidly to the body of the vehicle [5].

Since the navigation error caused by the bias of the inertial sensors increases with time, researchers proposed the rotation modulation method to reduce the impact on the SINS, which is installed on the vehicle through the turntable. The rotation modulation method modulates the bias of inertial sensors into a zero-mean and periodical form to inhibit the error propagation, and then, the localization precision of the SINS will be enhanced effectively. This kind of rotary strapdown inertial navigation system (RSINS) has been applied by the U.S. Navy since the 1960s [6-8]. However, the disadvantage of the rotation modulation is that it cannot modulate the installation error and the symmetric scale factor error of the inertial sensors, but it also produces instantaneous velocity error [8-10]. As a result, the design of the rotating angular rate has became a hotspot of rotation modulation.

The RSINS accuracy influenced by the non-uniform rotation angular rate is analyzed by Dr. Che [11]. The rotating scheme design method of the non-uniform rotating angular rate is proposed in [12]. However, until now, no one has been able to figure out an effective optimal rotating angular rate design method of the RSINS in theory. Therefore, in this article, a novel rotating angular rate design discipline, which will fill the gap, was proposed. We focused on the optimal design of the rotating angular rate utilizing the Laplace transform and the inverse Laplace transform in this article, and this paper is organized as follows. In Section 2, the principle of the rotation modulation and the inertial sensor error model are introduced. The design method of the rotating angular rate is proposed in Section 3. Simulations and experiments are carried out in Section 4 and 5, respectively, to verify the correctness of the design method.

## 2. Principle of the Rotation Modulation and the Error Model

### 2.1. Rotary Strapdown Inertial Navigation System

**Figure 1.**
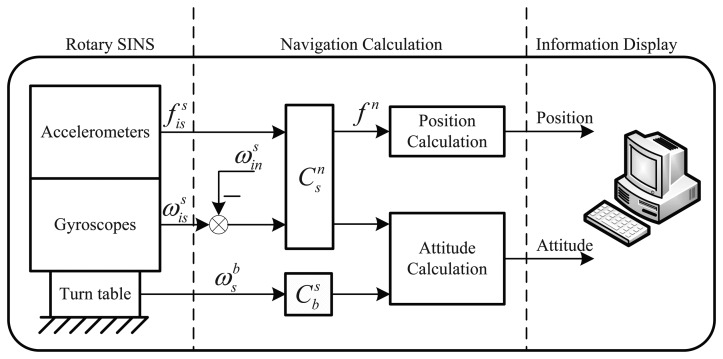
Schematic of the rotary strapdown inertial navigation system (RSINS).

As the SINS is installed on the vehicle through the turntable, the structure of the RSINS, which is shown as Figure 1, is different from the SINS. Since the middle of the last century, rotation modulation has been widely applied to the SINS [9,10].

Classified by the number of the rotating axes, the RSINS can be divided into single-axis RSINS and the dual-axis RSINS [13]. However, the purpose is the same, *i.e.* to compensate for the effect of inertial sensor errors without external information [14].

Additionally, we must say that any technique has its limitations, and rotation modulation is no exception. One fatal shortcoming of rotation modulation is that it will produce instantaneous velocity error that cannot be ignored during the rotating period, and the error exists in any rotating mode [8,15].

The essence of rotation modulation is the error self-compensation [16-18]. It modulates the inertial sensor errors in terms of periodical rotation. It is an effective method to enhance the navigation accuracy under low-accuracy sensors conditions [19-21]. The inertial sensor errors neither disappear nor change, but offset during the whole rotation period [21-24].

As the inertial measurement unit (IMU) connects with the vehicle indirectly through the turntable, a new frame, the sensor frame (*s* frame), is introduced in the RSINS. It is supposed that the body frame (*b* frame) and the navigation frame (*n* frame) are coherent. This means $C_{n}^{b}=I$ and $C_{b}^{s}=C_{n}^{s}$. The output of the inertial sensors is transformed from the *s* frame to the *n* frame by a direction cosine matrix. Suppose the *s* frame and *n* frame overlap at the initial time, and the IMU is controlled to rotate uniformly at a certain speed, *ω_r_,* along the vertical direction. The direction cosine matrix from the *s* frame to the *n* frame can be expressed by:

(1) $$C_{s}^{n}={\left(C_{n}^{s}\right)}^{T}={\left(C_{b}^{s}\right)}^{T}=\left[\begin{array}{ccc} \cos\omega_{r}t & -\sin\omega_{r}t & 0\\ \sin\omega_{r}t & \cos\omega_{r}t & 0\\ 0 & 0 & 1 \end{array}\right]$$

The modulated constant bias of the gyroscope in the *n* frame [*ɛ _E_*_1_ *ɛ_N_*_1_ *ɛ_U_*_1_]*^T^* can be described by the following equation.

(2) $$\left[\begin{array}{c} {ɛ}_{E1}\\ {ɛ}_{N1}\\ {ɛ}_{U1} \end{array}\right]=C_{s}^{n}\left[\begin{array}{c} {ɛ}_{x0}\\ {ɛ}_{y0}\\ {ɛ}_{z0} \end{array}\right]=\left[\begin{array}{c} {ɛ}_{x0}\cos\omega_{r}t-{ɛ}_{y0}\sin\omega_{r}t\\ {ɛ}_{x0}\sin\omega_{r}t+{ɛ}_{y0}\cos\omega_{r}t\\ {ɛ}_{z0} \end{array}\right]$$

where *ɛ_x_*_0_*, ɛ_y_*_0_*, ɛ_z_*_0_ denote the constant bias of the three orthogonal gyroscopes. From Equation (2), it is obviously known that the horizontal constant bias of the gyroscope can be modulated into periodic and zero-mean signals by periodically rotating the IMU. Analogously, it has the same result for the accelerometer. This technique can be utilized to modulate the inertial sensor error, which is vertical to the rotating axis, in order to enhance the positioning accuracy of the RSINS.

### 2.2. The Sensor Error Model of the RSINS

The inertial sensor error mainly includes the constant bias, the scale factor error and the installation error of the optical gyroscope and the constant bias of the accelerometer. In Section 2, we will focus on the relationship between the inertial sensor error and the performance of the RSINS.

#### 2.2.1. Constant Bias of Optical Gyroscopes and Accelerometers

The modulated constant bias of the gyroscope and the accelerometer in the *n* frame can be described by Equations (2) and (3), respectively.

(3) $$\left[\begin{array}{c} \nabla_{E}\\ \nabla_{N} \end{array}\right]=\left[\begin{array}{cc} \cos\omega_{r}t & -\sin\omega_{r}t\\ \sin\omega_{r}t & \cos\omega_{r}t \end{array}\right]\;\left[\begin{array}{c} \nabla_{x0}\\ \nabla_{y0} \end{array}\right]=\left[\begin{array}{c} \nabla_{x0}\cos\omega_{r}t-\nabla_{y0}\sin\omega_{r}t\\ \nabla_{y0}\sin\omega_{r}t+\nabla_{x0}\cos\omega_{r}t \end{array}\right]$$

where ∇*_x_*_0_ and ∇*_y_*_0_ are the constant bias of horizontal accelerometers.

#### 2.2.2. Scale Factor Error of Optical Gyroscopes

The equivalent bias of gyroscopes, *ε′*, caused by the scale factor asymmetric error of gyroscopes can be described by:

(4) $$\left[\begin{array}{c} {{ɛ}_{x}}^{\prime }\\ {{ɛ}_{y}}^{\prime }\\ {{ɛ}_{z}}^{\prime } \end{array}\right]=\left[\begin{array}{ccc} K_{gx} & 0 & 0\\ 0 & K_{gy} & 0\\ 0 & 0 & K_{gz} \end{array}\right]\;\left[\begin{array}{c} \omega_{x}\\ \omega_{y}\\ \omega_{z} \end{array}\right]=\left[\begin{array}{c} K_{gx}\cdot \omega_{ie}\cdot \cos L\cdot \sin\omega_{r}t\\ K_{gy}\cdot \omega_{ie}\cdot \cos L\cdot \cos\omega_{r}t\\ K_{gz}(\omega_{ie}\cdot \sin L+\omega_{r}) \end{array}\right]$$

where *K_gx_, K_gy_* and *K_gz_* denote the scale factor asymmetry errors of gyroscopes along the *x, y* and *z* axes, respectively; *ω_ie_* denotes the Earth's rotation rate; *L* denotes the local latitude; [*ω_x_ ω_y_ ω_z_*]*^T^* denotes the output of gyroscopes, and its theoretical value is:

(5) $$\left[\begin{array}{c} \omega_{x}\\ \omega_{y}\\ \omega_{z} \end{array}\right]=\left[\begin{array}{c} \omega_{ie}\cdot \cos L\cdot \sin\omega_{r}t\\ \omega_{ie}\cdot \cos L\cdot \cos\omega_{r}t\\ \omega_{ie}\cdot \sin L+\omega_{r} \end{array}\right]$$

Therefore, the modulated equivalent bias of gyroscopes, *ε′*′, in the *n* frame can be described by:

(6) $$\left[\begin{array}{c} {ɛ}_{E2}\\ {ɛ}_{N2}\\ {ɛ}_{U2} \end{array}\right]=\left[\begin{array}{ccc} \cos\omega_{r}t & -\sin\omega_{r}t & 0\\ \sin\omega_{r}t & \cos\omega_{r}t & 0\\ 0 & 0 & 1 \end{array}\right]\;\left[\begin{array}{c} {{ɛ}_{x}}^{\prime }\\ {{ɛ}_{y}}^{\prime }\\ {{ɛ}_{z}}^{\prime } \end{array}\right]=\left[\begin{array}{c} {{ɛ}_{x}}^{\prime }\cos\omega_{r}t-{{ɛ}_{y}}^{\prime }\sin\omega_{r}t\\ {{ɛ}_{y}}^{\prime }\cos\omega_{r}t-{{ɛ}_{x}}^{\prime }\sin\omega_{r}t\\ {{ɛ}_{z}}^{\prime } \end{array}\right]$$

#### 2.2.3. Installation Error of Optical Gyroscopes

**Figure 2.**
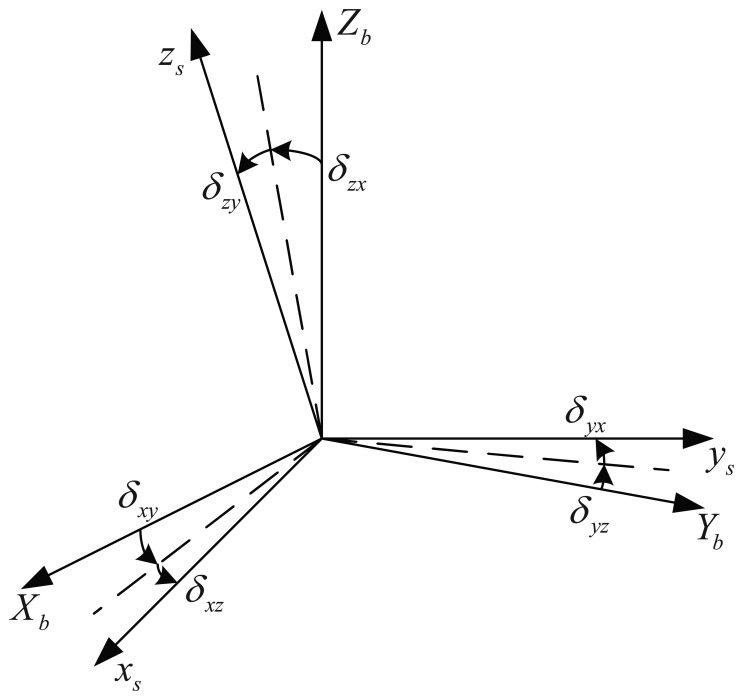
The geometrical relationship between *x_s_y_s_z_s_* and *X_b_Y_b_Z_b_*.

The inertial sensors should be installed orthogonally in theory. However, the actually *s* frame is not orthogonal and cannot coincide with the *b* frame, as shown in Figure 2. The small-angle *δ_ij_* is defined as the installation error between the *i* axis of the *s* frame and the *j* axis of the *b* frame. Therefore, the installation error matrix can be described as:

(7) $$\Delta C_{b}^{s}=\left[\begin{array}{ccc} 0 & -\delta_{xz} & \delta_{xy}\\ \delta_{yz} & 0 & -\delta_{yx}\\ -\delta_{yz} & \delta_{zx} & 0 \end{array}\right]$$

The equivalent bias of gyroscopes, ${ɛ}_{z}^{\prime \prime }$, caused by the installation error can be described by:

(8) $$\begin{array}{cc} \left[\begin{array}{c} {ɛ}_{x}^{\prime \prime }\\ {ɛ}_{y}^{\prime \prime }\\ {ɛ}_{z}^{\prime \prime } \end{array}\right] & =\left[\begin{array}{ccc} 0 & -\delta_{xz} & \delta_{xy}\\ \delta_{yz} & 0 & -\delta_{yx}\\ -\delta_{yz} & \delta_{zx} & 0 \end{array}\right]\;\left[\begin{array}{c} \omega_{x}\\ \omega_{y}\\ \omega_{z} \end{array}\right]\\ & =\left[\begin{array}{c} \delta_{xy}(\omega_{ie}\sin L+\omega_{r})-\delta_{xz}\omega_{ie}\cos L\cos\omega_{r}t\\ -\delta_{yx}(\omega_{ie}\sin L+\omega_{r})+\delta_{yz}\omega_{ie}\cos L\sin\omega_{r}t\\ \delta_{zx}\omega_{ie}\cos L\cos\omega_{r}t-\delta_{yz}\omega_{ie}\cos L\sin\omega_{r}t \end{array}\right] \end{array}$$

Therefore, the modulated equivalent bias of gyroscopes, *ε″*, in the *n* frame can be described by:

(9) $$\left[\begin{array}{c} {ɛ}_{E3}\\ {ɛ}_{N3}\\ {ɛ}_{U3} \end{array}\right]=\left[\begin{array}{ccc} \cos\omega_{r}t & -\sin\omega_{r}t & 0\\ \sin\omega_{r}t & \cos\omega_{r}t & 0\\ 0 & 0 & 1 \end{array}\right]\;\left[\begin{array}{c} {{ɛ}_{x}}^{\prime \prime }\\ {{ɛ}_{y}}^{\prime \prime }\\ {{ɛ}_{z}}^{\prime \prime } \end{array}\right]=\left[\begin{array}{c} {{ɛ}_{x}}^{\prime \prime }\cos\omega_{r}t-{{ɛ}_{y}}^{\prime \prime }\sin\omega_{r}t\\ {{ɛ}_{y}}^{\prime \prime }\cos\omega_{r}t+{{ɛ}_{x}}^{\prime \prime }\sin\omega_{r}t\\ {{ɛ}_{z}}^{\prime \prime } \end{array}\right]$$

#### 2.2.4. Error Model of Inertial Sensors in the RSINS

Concerning the study above, the error model of the RSINS can be described as

(10) $$\left[\begin{array}{c} \nabla_{E}\\ \nabla_{N}\\ 0\\ {ɛ}_{E}\\ {ɛ}_{N}\\ {ɛ}_{U} \end{array}\right]=\left[\begin{array}{c} \nabla_{E}\\ \nabla_{N}\\ 0\\ {ɛ}_{E1}+{ɛ}_{E2}+{ɛ}_{E3}\\ {ɛ}_{N1}+{ɛ}_{N2}+{ɛ}_{N3}\\ {ɛ}_{U1}+{ɛ}_{U2}+{ɛ}_{U3} \end{array}\right]=W_{1}(t)+W_{2}(t)+W_{3}(t)$$

where *W*_1_(*t*), *W*_2_(*t*) and *W*_3_(*t*) represent the constant bias error of inertial sensors, the scale factor error and the installation error of gyroscopes, respectively.

$$\begin{array}{cc} W_{1}(t)=\left[\begin{array}{c} \nabla_{x0}\cos\omega_{r}t-\nabla_{y0}\sin\omega_{r}t\\ \nabla_{x0}\sin\omega_{r}t+\nabla_{y0}\cos\omega_{r}t\\ 0\\ {ɛ}_{x0}\cos\omega_{r}t-{ɛ}_{y0}\sin\omega_{r}t\\ {ɛ}_{y0}\cos\omega_{r}t+{ɛ}_{x0}\sin\omega_{r}t\\ {ɛ}_{z0} \end{array}\right], & W_{2}(t)=\left[\begin{array}{c} 0\\ 0\\ 0\\ {ɛ}_{x}^{\prime }\cos\omega_{r}t-{ɛ}_{y}^{\prime }\sin\omega_{r}t\\ {ɛ}_{y}^{\prime }\cos\omega_{r}t+{ɛ}_{x}^{\prime }\sin\omega_{r}t\\ {ɛ}_{z}^{\prime } \end{array}\right],\\ W_{3}(t)=\left[\begin{array}{c} 0\\ 0\\ 0\\ {ɛ}_{x}^{\prime \prime }\cos\omega_{r}t-{ɛ}_{y}^{\prime \prime }\sin\omega_{r}t\\ {ɛ}_{y}^{\prime \prime }\cos\omega_{r}t-{ɛ}_{x}^{\prime \prime }\sin\omega_{r}t\\ {ɛ}_{z}^{\prime \prime } \end{array}\right] \end{array}$$

It can be seen from Equation (10) that the error model of the RSINS is a function that depends on not only the error of inertial sensors, but also the rotating angular rate. Therefore, the performance of the RSINS will be influenced by the rotating angular rate.

## 3. Optimal Design Method for the Rotating Angular Rate in the RSINS

The previous analysis shows that the error model of the RSINS bears on both the inertial sensor error and the rotating angular rate. Therefore, this section will investigate the relationship between the rotating angular rate and the RSINS performance.

### 3.1. The Velocity Error Equation of the RSINS

Based on the spherical Earth model, ignoring the movement of the vehicle, suppose the IMU rotates along the vertical axis at *ω_r_*; the error model of the RSINS can be described as:

(11) $$\dot{X}(t)=AX(t)+W_{1}(t)+W_{2}(t)+W_{3}(t)$$

where *X*(*t*)=[δ*V_E_* δ*V_N_* δ*L φ_E_ φ_n_ φ_U_*]*^t^* and:

$$\begin{array}{c} A=\left[\begin{array}{cc} A_{11} & A_{12}\\ A_{21} & A_{22} \end{array}\right];A_{11}=\left[\begin{array}{ccc} 0 & 2\omega_{ie} & 0\\ -2\omega_{ie} & 0 & 0\\ 0 & 1/R & 0 \end{array}\right];A_{12}=\left[\begin{array}{ccc} 0 & -g & 0\\ g & 0 & 0\\ 0 & 0 & 0 \end{array}\right];\\ A_{21}=\left[\begin{array}{ccc} 0 & -1/R & 0\\ 1/R & 0 & -\omega_{ie}\sin L\\ \tan L/R & 0 & \omega_{ie}\cos L \end{array}\right];A_{22}=\left[\begin{array}{ccc} 0 & \omega_{ie}\sin L & -\omega_{ie}\cos L\\ \omega_{ie}\sin L & 0 & 0\\ \omega_{ie}\cos L & 0 & 0 \end{array}\right] \end{array}$$

where *δV_E_* and *δV_N_* represent the velocity errors in the east and north, respectively; *δL* represents the error of the local latitude; *φ_E_, φ_N_* and *φ_U_* represent the misalignment angle of the east, north and up; *R* represents the radius of the Earth; *g* represents the gravitational acceleration. After the Laplace transform and ignoring the initial value, Equation (11) is transformed to the following format:

(12) $$X(s)={\left(sI-A\right)}^{-1}[W_{1}(s)+W_{2}(s)+W_{3}(s)]$$

Suppose M = (sI - A)^-1^ and $M={\left[M_{1}^{T}\;M_{2}^{T}\;M_{3}^{T}\;M_{4}^{T}\;M_{5}^{T}\;M_{6}^{T}\right]}^{T}$, where *M_j_* = [m*_ji_ m_j2_* … *m_j6_*] (*j* = 1, 2, …, 6), then:

$$\begin{array}{c} M_{1}=[\frac{s}{s^{2}+\omega_{s}^{2}}0*\frac{g\omega_{ie}\sin L\cdot s}{\left(s^{2}+\omega_{s}^{2})(s^{2}+\omega_{ie}^{2}\right)}-\frac{g(s^{2}+\omega_{ie}^{2}\cos^{2}L)}{\left(s^{2}+\omega_{s}^{2})(s^{2}+\omega_{ie}^{2}\right)}\frac{g\omega_{ie}^{2}\sin L\cos L}{\left(s^{2}+\omega_{s}^{2})(s^{2}+\omega_{ie}^{2}\right)}]\\ M_{2}=[0\frac{s}{s^{2}+\omega_{s}^{2}}*\frac{gs^{2}}{\left(s^{2}+\omega_{s}^{2})(s^{2}+\omega_{ie}^{2}\right)}-\frac{sg\omega_{ie}\sin L}{\left(s^{2}+\omega_{s}^{2})(s^{2}+\omega_{ie}^{2}\right)}-\frac{sg\omega_{ie}\cos L}{\left(s^{2}+\omega_{s}^{2})(s^{2}+\omega_{ie}^{2}\right)}]\\ M_{3}=[0-\frac{1}{R(s^{2}+\omega_{s}^{2})}*-\frac{\omega_{s}^{2}\cdot s}{\left(s^{2}+\omega_{s}^{2})(s^{2}+\omega_{ie}^{2}\right)}\frac{\omega_{s}^{2}\omega_{ie}\sin L}{\left(s^{2}+\omega_{s}^{2})(s^{2}+\omega_{ie}^{2}\right)}\frac{\omega_{s}^{2}\omega_{ie}\cos L}{\left(s^{2}+\omega_{s}^{2})(s^{2}+\omega_{ie}^{2}\right)}]\\ \begin{array}{c} M_{4}=[\frac{1}{R(s^{2}+\omega_{s}^{2})}-\frac{1}{R(s^{2}+\omega_{s}^{2})}*-\frac{s^{3}}{\left(s^{2}+\omega_{s}^{2})(s^{2}+\omega_{ie}^{2}\right)}\\ -\frac{s^{2}\omega_{ie}\sin L}{\left(s^{2}+\omega_{s}^{2})(s^{2}+\omega_{ie}^{2}\right)}-\frac{s^{2}\omega_{ie}\cos L}{\left(s^{2}+\omega_{s}^{2})(s^{2}+\omega_{ie}^{2}\right)}] \end{array}\\ M_{5}=[\frac{1}{R(s^{2}+\omega_{s}^{2})}0*-\frac{s^{2}\omega_{ie}\sin L}{\left(s^{2}+\omega_{s}^{2})(s^{2}+\omega_{ie}^{2}\right)}\frac{s(s^{2}+\omega_{ie}^{2}\cos^{2}L)}{\left(s^{2}+\omega_{s}^{2})(s^{2}+\omega_{ie}^{2}\right)}-\frac{s\omega_{ie}^{2}\sin L\cos L)}{\left(s^{2}+\omega_{s}^{2})(s^{2}+\omega_{ie}^{2}\right)}]\\ \begin{array}{c} M_{6}=[\frac{\tan L}{R(s^{2}+\omega_{s}^{2})}0*\frac{\omega_{ie}\cos Ls^{2}+\omega_{ie}\omega_{s}^{2}\sec L}{\left(s^{2}+\omega_{s}^{2})(s^{2}+\omega_{ie}^{2}\right)}\\ \frac{s(\omega_{s}^{2}\tan L-\omega_{ie}^{2}\sin L\cos L)}{\left(s^{2}+\omega_{s}^{2})(s^{2}+\omega_{ie}^{2}\right)}\frac{s(s^{2}+\omega_{ie}^{2}\sin^{2}L+\omega_{s}^{2})}{\left(s^{2}+\omega_{s}^{2})(s^{2}+\omega_{ie}^{2}\right)}] \end{array} \end{array}$$

As the values of *m_j_*_3_(*j* = 1, 2, …, 6) do not affect *X*(*s*), the elements of the third column are represented with ‘*’. *W_i_*(*s*), the expression of *W_i_*(*t*) in the frequency domain, can be expressed as:

$$\begin{array}{ccc} W_{1}(s)=\left[\begin{array}{c} \frac{s\nabla_{x0}-\omega_{r}\nabla_{y0}}{s^{2}+\omega_{r}^{2}}\\ \frac{\omega_{r}\nabla_{x0}+s\nabla_{y0}}{s^{2}+\omega_{r}^{2}}\\ 0\\ \frac{s{ɛ}_{x0}-\omega_{r}{ɛ}_{y0}}{s^{2}+\omega_{r}^{2}}\\ \frac{s{ɛ}_{y0}+\omega_{r}{ɛ}_{x0}}{s^{2}+\omega_{r}^{2}}\\ \frac{{ɛ}_{z0}}{s} \end{array}\right], & W_{1}(s)=\left[\begin{array}{c} 0\\ 0\\ 0\\ \frac{s{ɛ}_{x}^{\prime }(s)-\omega_{r}{ɛ}_{y}^{\prime }(s)}{s^{2}+\omega_{r}^{2}}\\ \frac{s{ɛ}_{y}^{\prime }(s)+\omega_{r}{ɛ}_{x}^{\prime }(s)}{s^{2}+\omega_{r}^{2}}\\ \frac{{ɛ}_{z}^{\prime }}{s} \end{array}\right], & W_{3}(s)=\left[\begin{array}{c} 0\\ 0\\ 0\\ \frac{s{ɛ}_{x}^{\prime \prime }(s)-\omega_{r}{ɛ}_{y}^{\prime \prime }(s)}{s^{2}+\omega_{r}^{2}}\\ \frac{s{ɛ}_{y}^{\prime \prime }(s)+\omega_{r}{ɛ}_{x}^{\prime \prime }(s)}{s^{2}+\omega_{r}^{2}}\\ \frac{{ɛ}_{z}^{\prime \prime }}{s} \end{array}\right] \end{array}$$

where:

$$\begin{array}{c} {ɛ}_{x}^{\prime }(s)=\frac{K_{gx}\omega_{ie}\cdot \cos L\cdot \omega_{r}}{s^{2}+\omega_{r}^{2}};{ɛ}_{y}^{\prime }(s)=\frac{K_{gy}\omega_{ie}\cdot \cos L\cdot s}{s^{2}+\omega_{r}^{2}};{ɛ}_{z}^{\prime }(s)=\frac{K_{gz}(\omega_{ie}\sin L+\omega_{r})}{s};\\ {ɛ}_{y}^{\prime \prime }(s)=\frac{\delta_{yz}(\omega_{ie}\sin L+\omega_{r})}{s}-\frac{\delta_{xz}\omega_{ie}\cdot \cos L\cdot s}{s^{2}+\omega_{r}^{2}};\\ {ɛ}_{y}^{\prime \prime }(s)=\frac{\delta_{xy}(\omega_{ie}\cos L\cdot \omega_{r})}{s^{2}+\omega_{r}^{2}}-\frac{\delta_{yx}(\omega_{ie}\cdot \sin L+\omega_{r})}{s};\\ {ɛ}_{z}^{\prime \prime }(s)=\frac{\delta_{zx}\omega_{ie}\cdot \cos L\cdot s}{s^{2}+\omega_{r}^{2}}-\frac{\delta_{zy}\omega_{ie}\cdot \cos L\cdot \omega_{r})}{s^{2}+\omega_{r}^{2}}; \end{array}$$

Therefore, the velocity error of the east and north in the frequency domain can be expressed as:

(13) $$\{\begin{array}{c} \delta V_{E}(s)=M_{1}\cdot W_{1}(s)+M_{1}\cdot W_{2}(s)+M_{1}\cdot W_{3}(s)+M_{1}\cdot W_{4}(s)\\ \delta V_{N}(s)=M_{2}\cdot W_{1}(s)+M_{2}\cdot W_{2}(s)+M_{2}\cdot W_{3}(s)+M_{2}\cdot W_{4}(s) \end{array}$$

where *M_i_* · *W*_1_(*s*)(*i* = 1,2), *M_i_* · *W_2_*(*s*)(*i* = 1,2), *M_i_* · *W*_3_(*s*)(*i* = 1,2) and *M_i_* · *W_4_*(*s*)(*i* = 1,2) represent the velocity error caused by the constant bias of gyroscopes, the velocity error caused by the constant bias of accelerometers, the velocity error caused by the gyroscope scale factor error and the velocity error caused by the gyroscope installation error, respectively.

#### 3.1.1. Velocity Error Caused by the Constant Bias of Gyroscopes

The east velocity error caused by the gyroscope constant bias can be expressed in the frequency domain as:

(14) $$\begin{array}{cc} \delta V_{E}^{1}(s) & =M_{1}\cdot W_{1}(s)\\ & =\frac{g\omega_{ie}^{2}\sin L\cdot \cos L{ɛ}_{z0}}{s(s^{2}+\omega_{s}^{2})(s^{2}+\omega_{ie}^{2})}+\frac{sg\omega_{ie}\sin L(s{ɛ}_{x0}-\omega_{r}{ɛ}_{y0})}{\left(s^{2}+\omega_{s}^{2})(s^{2}+\omega_{ie}^{2})(s^{2}+\omega_{r}^{2}\right)}-\frac{g\omega_{ie}\sin Ls(s{ɛ}_{y0}-\omega_{r}{ɛ}_{x0})}{\left(s^{2}+\omega_{r}^{2})(s^{2}+\omega_{ie}^{2})(s^{2}+\omega_{s}^{2}\right)} \end{array}$$

After the inverse Laplace transform and the simplification, we can get the east velocity error caused by the gyroscope constant bias in the time domain shown as:

(15) $$\delta V_{E}^{1}(t)=k_{11}\cos\omega_{s}t+k_{12}\sin\omega_{s}t+k_{13}\cos\omega_{r}t+k_{14}\sin\omega_{r}t+k_{15}\cos\omega_{ie}t+k_{16}$$

The amplitude of the east velocity error is expressed as:

(16) $$A_{E1}(\omega_{r})=\sqrt{k_{11}^{2}+k_{12}^{2}+k_{13}^{2}+k_{14}^{2}+k_{15}^{2}+k_{16}^{2}}$$

where:

$$\begin{array}{l} \begin{array}{cccc} k_{11}=\frac{g\omega_{ie}^{2}\cos L\sin L}{\omega_{s}^{4}}{ɛ}_{z0}; & k_{12}=-\frac{g}{\omega_{r}\omega_{s}}{ɛ}_{z0}; & k_{13}=-\frac{g}{\omega_{r}^{2}}{ɛ}_{x0}; & k_{14}=-\frac{g}{\omega_{r}^{2}}{ɛ}_{y0}; \end{array}\\ \begin{array}{cc} k_{15}=\frac{g\sin L\cos L}{\omega_{s}^{2}}{ɛ}_{z0}; & k_{16}=\frac{g\sin L\cos L}{\omega_{s}^{2}}{ɛ}_{z0}. \end{array} \end{array}$$

The north velocity error caused by the gyroscope constant bias can be expressed in the frequency domain as:

(17) $$\delta V_{N}^{1}(s)=\frac{gs^{2}(s{ɛ}_{x0}-\omega_{r}{ɛ}_{y0})}{\left(s^{2}+\omega_{r}^{2})(s^{2}+\omega_{s}^{2})(s^{2}+\omega_{ie}^{2}\right)}-\frac{g\omega_{ie}\cos Ls{ɛ}_{z0}}{s(s^{2}+\omega_{s}^{2})(s^{2}+\omega_{ie}^{2})}-\frac{g\omega_{ie}\sin Ls(s{ɛ}_{y0}-\omega_{r}{ɛ}_{x0})}{\left(s^{2}+\omega_{r}^{2})(s^{2}+\omega_{ie}^{2})(s^{2}+\omega_{s}^{2}\right)}$$

After the inverse Laplace transform and simplification, one can get the north velocity error caused by the gyroscope constant bias in the time domain as:

(18) $$\delta V_{N}^{1}=k_{21}\cos\omega_{s}t+k_{22}\sin\omega_{s}t+k_{23}\cos\omega_{r}t+k_{24}\sin\omega_{r}t+k_{25}\cos\omega_{ie}t+k_{26}\sin\omega_{ie}t$$

The amplitude of the north velocity error is expressed as:

(19) $$A_{N1}(\omega_{r})=\sqrt{k_{21}^{2}+k_{22}^{2}+k_{23}^{2}+k_{24}^{2}+k_{25}^{2}+k_{26}^{2}}$$

where:

$$\begin{array}{l} \begin{array}{cccc} k_{21}=\frac{g\omega_{ie}\sin L}{\omega_{s}^{2}\omega_{r}}{ɛ}_{x0}; & k_{22}=\frac{g\omega_{ie}\cos L}{\omega_{s}^{3}}{ɛ}_{z0}; & k_{23}=-\frac{1}{\omega_{r}^{2}}{ɛ}_{x0}; & k_{24}=\frac{1}{\omega_{r}^{2}}{ɛ}_{y0}; \end{array}\\ \begin{array}{cc} k_{25}=\frac{\omega_{ie}g\sin L}{\omega_{r}\omega_{s}^{2}}{ɛ}_{x0}; & k_{26}=-\frac{g\cos L}{\omega_{s}^{2}}{ɛ}_{z0} \end{array} \end{array}$$

**Figure 3.**
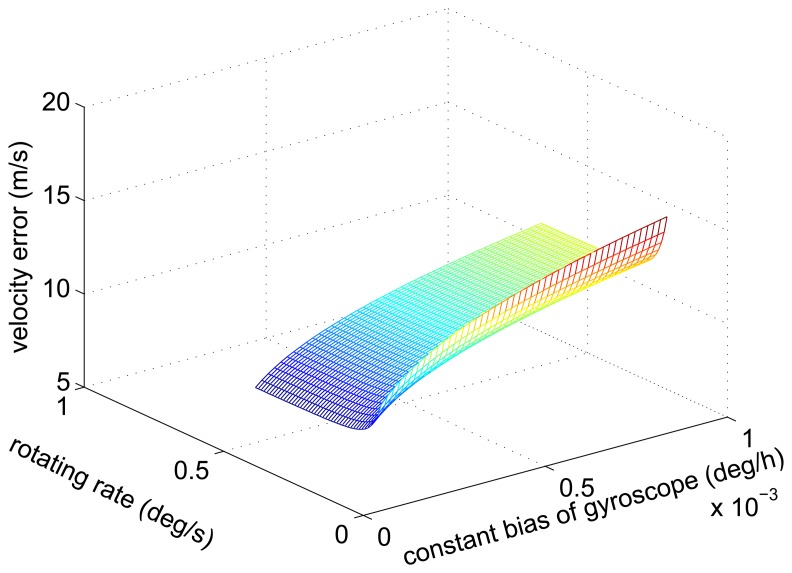
The relationship curve of the amplitude of the velocity error, the constant bias of the gyroscope and the rotating angular rate.

It is known that the amplitude of the velocity error caused by the gyroscope constant bias decreases with the increase of the rotating angular rate from Equations (16) and (19). Figure 3 shows the simulation results.

#### 3.1.2. Velocity Error Caused by the Constant Bias of Accelerometers

The east velocity error caused by the accelerometer constant bias can be expressed in the frequency domain as:

(20) $$\delta V_{E}^{2}(s)=M_{1}\cdot W_{2}(s)=\frac{s(s\nabla_{x0}-\omega_{r}\nabla_{y0})}{\left(s^{2}+\omega_{s}^{2})(s^{2}+\omega_{r}^{2}\right)}$$

After the inverse Laplace transform and simplification, we get the east velocity error caused by the accelerometer constant bias in the time domain and shown as:

(21) $$\delta V_{E}^{2}(t)=k_{31}\cos\omega_{s}t+k_{32}\sin\omega_{s}t+k_{33}\cos\omega_{r}t+k_{34}\sin\omega_{r}t$$

The amplitude of the east velocity error is expressed as:

(22) $$A_{E2}(\omega_{r})=\sqrt{k_{31}^{2}+k_{32}^{2}+k_{33}^{2}+k_{34}^{2}}$$

where:

$$\begin{array}{cccc} k_{31}=-\frac{1}{\omega_{r}}\nabla_{y0}; & k_{32}=-\frac{\omega_{s}}{\omega_{r}^{2}}\nabla_{x0}; & k_{33}=\frac{1}{\omega_{r}}\nabla_{x0}; & k_{34}=\frac{1}{\omega_{r}}\nabla_{y0} \end{array}$$

The north velocity error caused by the accelerometer constant bias can be expressed in the frequency domain as:

(23) $$\delta V_{N}^{2}(s)=\frac{s(\omega_{r}\nabla_{x0}+s\nabla_{y0})}{\left(s^{2}+\omega_{s}^{2})(s^{2}+\omega_{r}^{2}\right)}$$

After the inverse Laplace transform and simplifying Equation (23), we get the east velocity error caused by the accelerometer constant bias in the time domain as:

(24) $$\delta V_{N}^{2}(t)=k_{41}\sin\omega_{r}t+k_{42}\sin\omega_{s}t-k_{43}\cos\omega_{r}t-k_{44}\sin\omega_{s}t$$

The amplitude of the east velocity error is expressed as:

(25) $$A_{N2}(\omega_{r})=\sqrt{k_{41}^{2}+k_{42}^{2}+k_{43}^{2}+k_{44}^{2}}$$

where:

$$\begin{array}{cccc} k_{41}=\frac{\nabla_{y0}}{\omega_{r}}; & k_{42}=\frac{\nabla_{x0}}{\omega_{r}}; & k_{43}=-\frac{\nabla_{x0}}{\omega_{r}}; & k_{44}=-\frac{\omega_{s}\nabla_{y0}}{\omega_{r}^{2}} \end{array}$$

**Figure 4.**
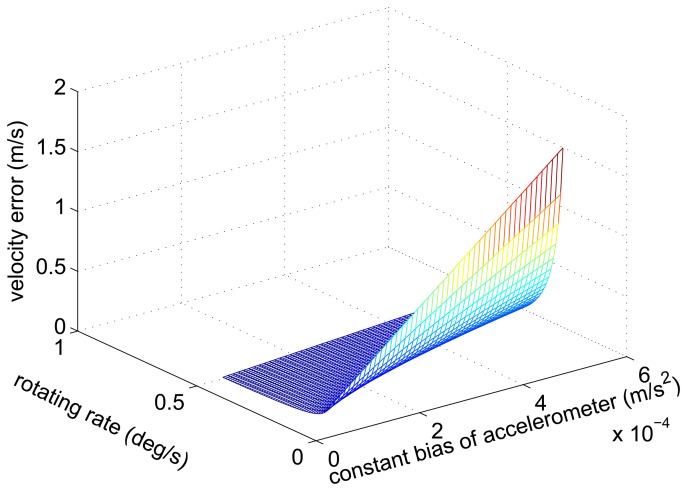
The relationship curve of the amplitude of the velocity error, the constant bias of the accelerometer and the rotating angular rate.

The conclusion is obtained from Equations (22) and (25) that the amplitude of the velocity error caused by the accelerometer constant bias decreases with the increase of the rotating angular rate. Figure 4 shows the simulation results.

#### 3.1.3. Velocity Error Caused by the Scale Factor Error

The east velocity error caused by the scale factor error can be expressed in the frequency domain as:

(26) $$\begin{array}{cc} \delta V_{E}^{3}(s) & =M_{1}\cdot W_{3}(s)\\ & \begin{array}{c} =\frac{g\omega_{r}\omega_{ie}\cos L(s^{2}\omega_{ie}\sin L-s^{2}\omega_{r}-\omega_{r}\omega_{ie}^{2}\cos^{2}L)}{(s^{2}+\omega_{s}^{2})(s^{2}+\omega_{ie}^{2}){\left(s^{2}+\omega_{r}^{2}\right)}^{2}}K_{gx}\\ -\frac{s^{2}g\omega_{ie}\cos L(s^{2}+\omega_{r}\omega_{ie}\sin L+\omega_{ie}^{2}\cos^{2}L)}{(s^{2}+\omega_{s}^{2})(s^{2}+\omega_{ie}^{2}){\left(s^{2}+\omega_{r}^{2}\right)}^{2}}K_{gy}+\frac{g\omega_{ie}^{2}\sin L\cos L(\omega_{ie}\sin L+\omega_{r})}{s^{2}(s^{2}+\omega_{s}^{2})(s^{2}+\omega_{ie}^{2})}K_{gz} \end{array} \end{array}$$

After the inverse Laplace transform and simplifying Equation (26), we get the east velocity error caused by the scale factor error in the time domain as:

(27) $$\delta V_{E}^{3}(t)=k_{51}\sin\omega_{r}t+k_{52}\sin\omega_{s}t+k_{53}\sin\omega_{ie}t+k_{54}t$$

The amplitude of the east velocity error is expressed as:

(28) $$A_{E3}(\omega_{r})=\sqrt{k_{51}^{2}+k_{52}^{2}+k_{53}^{2}+k_{54}^{2}}$$

where:

$$\begin{array}{l} \begin{array}{ccc} k_{51}=\frac{g\omega_{ie}^{2}\sin L\cos L}{\omega_{r}\omega_{s}^{5}}K_{gz}; & k_{52}=\frac{g\omega_{ie}\cos L}{\omega_{r}^{3}}K_{gx}; & k_{53}=\frac{g\omega_{r}\sin L\cos L}{\omega_{r}^{2}}K_{gz}; \end{array}\\ k_{54}=\frac{g\omega_{r}\sin L\cos L}{\omega_{r}^{2}}K_{gz} \end{array}$$

The north velocity error caused by the scale factor error can be expressed in the frequency domain as:

(29) $$\begin{array}{cc} \delta V_{N}^{3}(s) & =M_{2}\cdot W_{3}(s)\\ & \begin{array}{l} =\frac{g\omega_{ie}\cos L\omega_{r}s(s^{2}+\omega_{r}\omega_{ie}\sin L)}{(s^{2}+\omega_{s}^{2})(s^{2}+\omega_{ie}^{2}){\left(s^{2}+\omega_{r}^{2}\right)}^{2}}K_{gx}-\frac{s^{3}g\omega_{ie}\cos L(\omega_{r}+\omega_{ie}\sin L)}{(s^{2}+\omega_{s}^{2})(s^{2}+\omega_{ie}^{2}){\left(s^{2}+\omega_{r}^{2}\right)}^{2}}K_{gy}\\ -\frac{g\omega_{ie}\cos L(\omega_{ie}\sin L+\omega_{r})}{s(s^{2}+\omega_{s}^{2})(s^{2}+\omega_{ie}^{2})}K_{gz} \end{array} \end{array}$$

After the inverse Laplace transform and the simplification, we get the east velocity error caused by the scale factor error in the time domain as:

(30) $$\delta V_{N}^{3}(t)=k_{61}\cos\omega_{s}t+k_{62}\cos\omega_{r}t+k_{63}$$

The amplitude of the east velocity error is expressed as:

(31) $$A_{N3}(\omega_{r})=\sqrt{k_{61}^{2}+k_{62}^{2}+k_{63}^{2}}$$

where:

$$\begin{array}{ccc} k_{61}=-\frac{g\omega_{ie}^{2}\omega_{r}\cos L}{\omega_{s}^{4}}K_{gz}; & k_{62}=\frac{g\omega_{r}\cos L}{\omega_{s}^{2}}K_{gz}; & k_{63}=\frac{g\omega_{r}\cos L}{\omega_{s}^{2}}K_{gz} \end{array}$$

**Figure 5.**
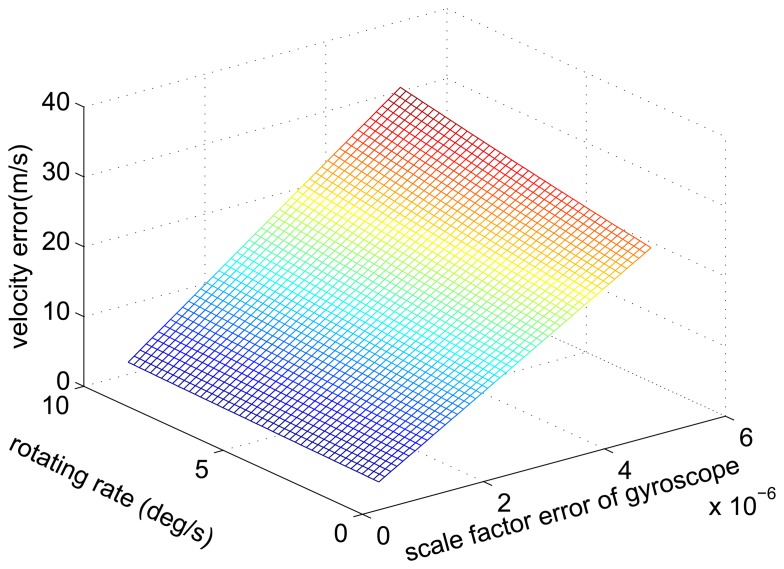
The relationship curve of the amplitude of the velocity error, the scale factor error and the rotating angular rate.

From Equations (28) and (31), we can draw a conclusion that the amplitude of the velocity error caused by the accelerometer constant bias increases with the increase of the rotating angular rate. Additionally, we plot the relationship between the amplitude of the velocity error, the scale factor error and the rotating angular rate as Figure 5.

#### 3.1.4. Velocity Error Caused by the Installation Error

The east velocity error caused by the installation error can be expressed in the frequency domain as:

(32) $$\begin{array}{ll} \delta V_{E}^{4}(s) & =M_{1}\cdot W_{4}(s)\\ & \begin{array}{l} =\frac{\left[g\omega_{ie}\sin Ls^{2}-g(s^{2}+\omega_{ie}^{2}\cos^{2}L)\omega_{r}](\omega_{ie}\sin L+\omega_{r}\right)}{s(s^{2}+\omega_{s}^{2})(s^{2}+\omega_{ie}^{2})(s^{2}+\omega_{r}^{2})}\delta_{xy}\\ -\frac{\omega_{ie}\cos Ls[g\omega_{ie}\sin Ls^{2}-g(s^{2}+\omega_{ie}^{2}\cos^{2}L)\omega_{r}]}{(s^{2}+\omega_{s}^{2})(s^{2}+\omega_{ie}^{2}){\left(s^{2}+\omega_{r}^{2}\right)}^{2}}\delta_{xz}\\ -\frac{\omega_{ie}\cos L\omega_{r}[g\omega_{ie}\sin Ls\omega_{r}+g(s^{2}+\omega_{ie}^{2}\cos^{2}L)s]}{(s^{2}+\omega_{s}^{2})(s^{2}+\omega_{ie}^{2}){\left(s^{2}+\omega_{r}^{2}\right)}^{2}}\delta_{yz}\\ +\frac{\left(\omega_{ie}\sin L+\omega_{r})[g\omega_{ie}\sin Ls\omega_{r}+g(s^{2}+\omega_{ie}^{2}\cos^{2}L)s\right]}{s(s^{2}+\omega_{s}^{2})(s^{2}+\omega_{ie}^{2})(s^{2}+\omega_{r}^{2})}\delta_{yx}\\ +\frac{g\omega_{ie}^{2}\sin L\cos^{2}L}{\left(s^{2}+\omega_{s}^{2})(s^{2}+\omega_{ie}^{2})(s^{2}+\omega_{r}^{2}\right)}\delta_{zx}-\frac{g\omega_{ie}^{3}\sin L\cos^{2}L\omega_{r}}{s(s^{2}+\omega_{s}^{2})(s^{2}+\omega_{ie}^{2})(s^{2}+\omega_{r}^{2})}\delta_{zy} \end{array} \end{array}$$

After the inverse Laplace transform and simplification, we get the east velocity error caused by the installation error in the time domain as:

(33) $$\delta V_{E}^{4}(t)=k_{71}\sin\omega_{r}t+k_{72}\cos\omega_{r}t+k_{73}\cos\omega_{s}t+k_{74}\sin\omega_{ie}t+k_{75}\cos\omega_{ie}t+k_{76}$$

The amplitude of the east velocity error is expressed as:

(34) $$A_{E4}(\omega_{r})=\sqrt{k_{71}^{2}+k_{72}^{2}+k_{73}^{2}+k_{74}^{2}+k_{75}^{2}+k_{76}^{2}}$$

where:

$$\begin{array}{c} \begin{array}{ccc} k_{71}=-\frac{g\delta_{yx}}{\omega_{r}^{2}}; & k_{72}=-\frac{g\delta_{xy}}{\omega_{r}^{2}}; & k_{73}=-\frac{g\omega_{ie}\sin L\cos^{2}L\delta_{zy}}{\omega_{s}^{4}\omega_{r}}; \end{array}\\ \begin{array}{ccc} k_{74}=\frac{g\sin L\delta_{yx}}{\omega_{s}^{2}}; & k_{75}=-\frac{g\sin L\delta_{xy}}{\omega_{s}^{2}}; & k_{76}=-\frac{g\sin L\delta_{yx}}{\omega_{s}^{2}\omega_{r}^{5}} \end{array} \end{array}$$

The north velocity error caused by the installation error can be expressed in the frequency domain as:

(35) $$\begin{array}{ll} \delta V_{N}^{4}(s) & =M_{2}\cdot W_{4}(s)\\ & \begin{array}{c} =\frac{g(s^{2}-\omega_{r}\omega_{ie}\sin L)(\omega_{ie}\sin L+\omega_{r})}{\left(s^{2}+\omega_{s}^{2})(s^{2}+\omega_{ie}^{2})(s^{2}+\omega_{r}^{2}\right)}\delta_{xy}-\frac{s^{2}g\omega_{ie}\cos L(s^{2}-\omega_{r}\omega_{ie}\sin L)}{(s^{2}+\omega_{s}^{2})(s^{2}+\omega_{ie}^{2}){\left(s^{2}+\omega_{r}^{2}\right)}^{2}}\delta_{xz}\\ -\frac{(\omega_{r}+\omega_{ie}\sin L)s^{2}g\omega_{ie}\cos L\omega_{r}}{(s^{2}+\omega_{s}^{2})(s^{2}+\omega_{ie}^{2}){\left(s^{2}+\omega_{r}^{2}\right)}^{2}}\delta_{yz}+\frac{gs{\left(\omega_{r}+\omega_{ie}\sin L\right)}^{2}}{\left(s^{2}+\omega_{s}^{2})(s^{2}+\omega_{ie}^{2})(s^{2}+\omega_{r}^{2}\right)}\delta_{yx}\\ -\frac{sg\omega ie^{2}\cos^{2}L}{\left(s^{2}+\omega_{s}^{2})(s^{2}+\omega_{ie}^{2})(s^{2}+\omega_{r}^{2}\right)}\delta_{zx}+\frac{g\omega_{r}\omega_{ie}^{2}\cos^{2}L}{\left(s^{2}+\omega_{s}^{2})(s^{2}+\omega_{ie}^{2})(s^{2}+\omega_{r}^{2}\right)}\delta_{zy} \end{array} \end{array}$$

After the inverse Laplace transform and simplification, we get the north velocity error caused by the installation error in the time domain as:

(36) $$\delta V_{N}^{4}(t)=k_{81}\cos\omega_{r}t+k_{82}\cos\omega_{s}t+k_{83}\cos\omega_{i}et+k_{84}\sin\omega_{r}t\cdot t$$

The amplitude of the north velocity error is expressed as:

(37) $$A_{N4}(\omega_{r})=\sqrt{k_{81}^{2}+k_{82}^{2}+k_{83}^{2}+k_{84}^{2}}$$

where:

$$\begin{array}{cccc} k_{81}=\frac{2g\delta_{yx}}{\omega_{r}^{4}}; & k_{82}=-\frac{g\delta_{yx}}{\omega_{r}^{2}\omega_{s}^{2}}; & k_{83}=-\frac{g\delta_{yx}}{\omega_{r}^{2}\omega_{s}^{2}}; & k_{84}=-\frac{g\delta_{yx}}{2\omega_{r}^{2}} \end{array}$$

**Figure 6.**
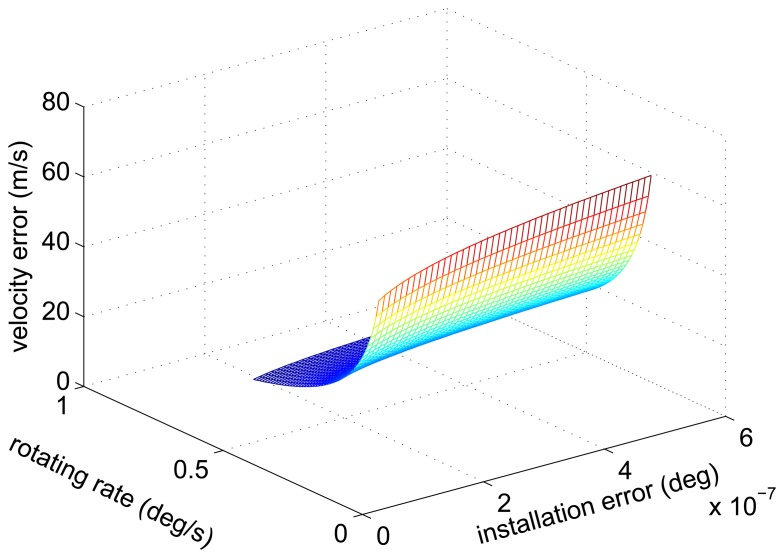
The relationship curve of the amplitude of the velocity error, the installation error and the rotating angular rate.

From Equations (34) and (37), we can draw a conclusion that the amplitude of the velocity error caused by the installation error increase with the increase of rotating angular rate. Additionally, we plot the relationship between the amplitude of the velocity error, the installation error and the rotating angular rate as Figure 6.

### 3.2. Optimal Design Method for the Angular Rate

Section 3.1 is focused on the relationship between the velocity error, three different kinds of inertial sensors errors and the rotating angular rate. In this part, we will propose the optimal design method of the rotating angular rate.

When the RSINS rotates at a constant speed, the amplitude of the velocity error can be expressed as:

(38) $$A(\omega_{r})=\sqrt{A_{1}^{2}(\omega_{r})+A_{2}^{2}(\omega_{r})+A_{3}^{2}(\omega_{r})+A_{4}^{2}(\omega_{r})}$$

where $\begin{array}{cc} A_{j}(\omega_{r})=\sqrt{A_{Ej}^{2}(\omega_{r})+A_{Nj}^{2}(\omega_{r})} & \left(j=1,\cdots ,4\right) \end{array}$.

From the Equation (38), it is known that the amplitude of the velocity error can be expressed as a function of the inertial sensor error and the rotating angular rate. Therefore, for the purpose of ensuring the minimum of the velocity error amplitude, the rotating angular rate should match the error of the inertial sensors. In order to verify and determine this matching relationship, we will design three groups of inertial sensor performances and calculate the optimal rotating angular rate of each group with MATLAB. Taking “MARINS”, a high-precision INS manufactured by IXBlue, as an example, its positioning accuracy is 1 nm/24 h. It can be inferred that the constant bias and the scale factor error of its gyroscopes are lower than 0.01 °/h and 1 × 10^-6^ (°/h)^-1^, respectively. On the other hand, considering the real INS, the constant bias accelerometer is lower than 9.8 × 10^-4^ m/s^2^, and the installation error of gyroscopes is lower than 1 × 10^-9^ °/h. Therefore, on the basis of the above, the simulation parameters are set as Tables 1-3, respectively.

**Figure 7.**
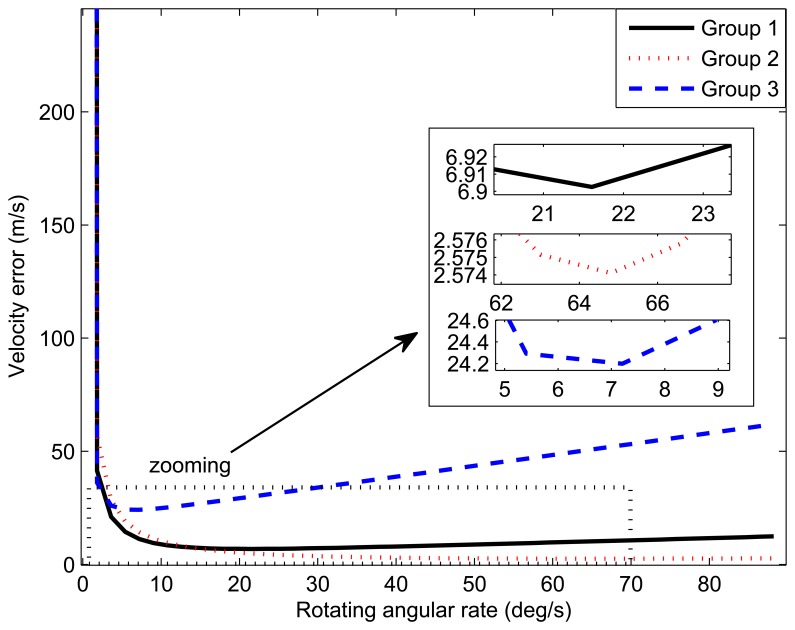
The relationship curves between the velocity errors and the rotating rates.

Figure 7 depicts the relationship between the amplitude of the velocity error and the rotating angular rate by MATLAB. The value of the horizontal axis corresponding to the minimum of the curve is the optimal rotating angular rate. The optimal rotating angular rates of these three groups are summarized in Table 4.

## 4. Numerical Simulations

**Figure 8.**
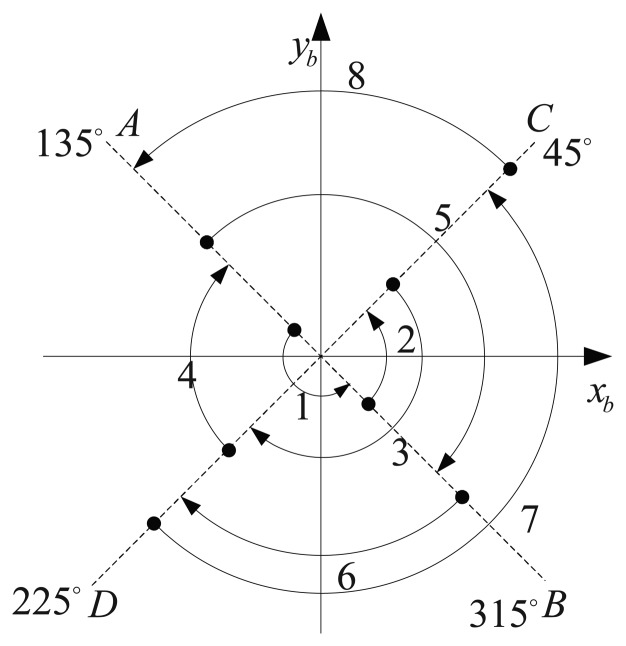
IMU rotating scheme.

The optimal design method of the rotating angular rate is proposed in Section 3. The theoretical optimal rotating angular rates corresponding to three different groups of inertial sensor errors are obtained in terms of this method. In order to verify the correctness of the former conclusions, simulations are carried out in this section. The rotating scheme is proposed as shown in Figure 8. The turntable rotates the IMU back and forth along the azimuth axis through four orthogonal positions. The dwell time in each position is 45 s [25].

The performances of inertial sensors are described in Tables 1, 2, and 3. The rotating angular rates are designed as 7.2 °/s, 21.6 °/s, 45 °/s and 64.8 °/s; as 21.6 °/s, 64.8 °/s and 7.2 °/s are the optimal rotating rate of Groups 1-3, respectively, and 45 °/s is chosen for comparison.

**Figure 9.**
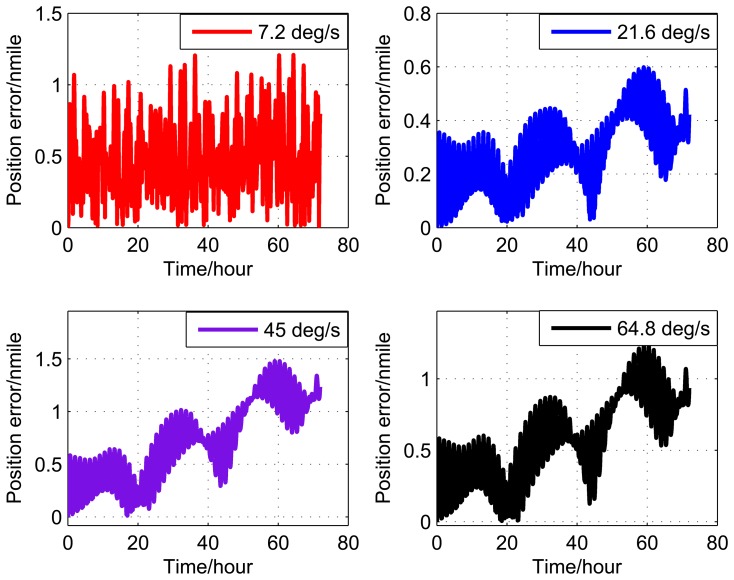
Simulation results of Group 1.

**Figure 10.**
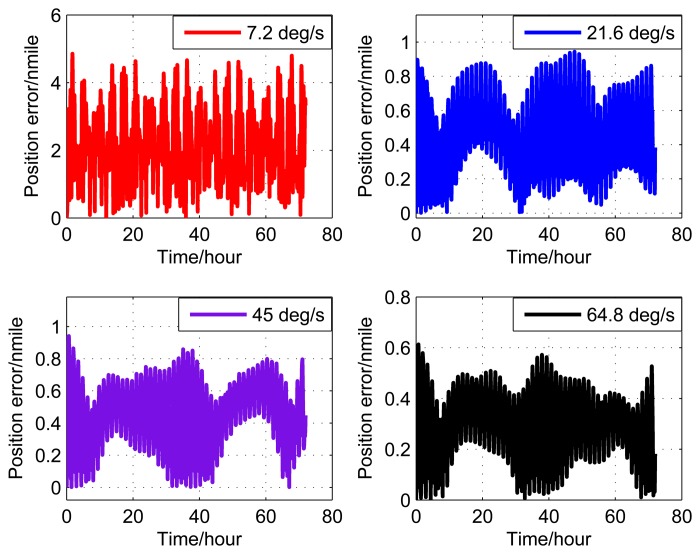
Simulation results of Group 2.

**Figure 11.**
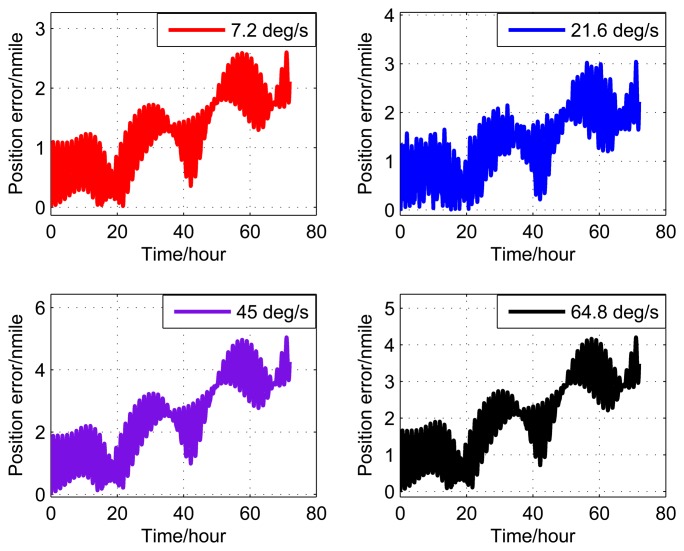
Simulation results of Group 3.

Figures 9, 10, and 11 show the simulation results of the position error of different groups. The simulation duration is 72 h.

Tables 5, 6, and 7 summarize the simulation results.

It can be seen from Figure 9 and Table 5 that the position error of Group 1 with a rotating angular rate of 21.6 °/s is lower than the other rotating angular rates. From Figure 10 and Table 6, it can be seen that the position error of Group 2 with a rotating angular rate of 64.8 °/s is lower than the other rotating angular rates. From Figure 10 and Table 6, we can see that the position error of Group 3 with a rotating angular rate of 7.2 °/s is lower than the other rotating angular rates. The simulation results are in accordance with the conclusions of Section 3.

## 5. Experimental Study

**Figure 12.**
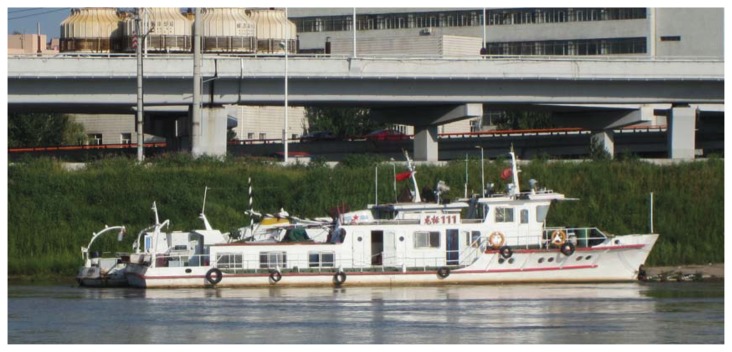
Experimental ship.

**Figure 13.**
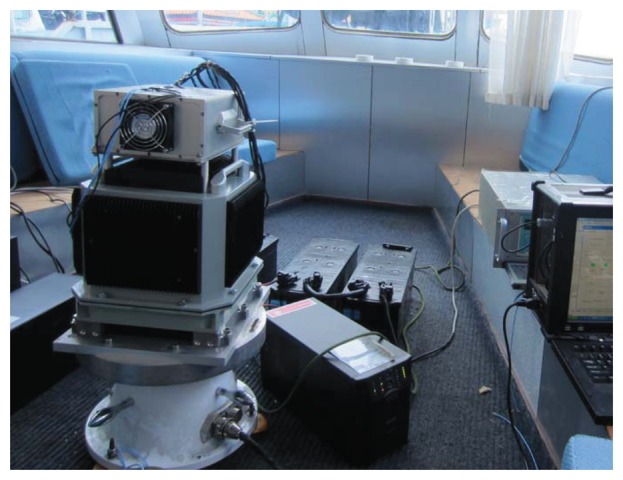
Single-axis RSINS.

In order to verify the effectiveness of the method, experiments were done in Songhuajiang River in Harbin, as shown in Figure 12. Experimental equipment, as shown in Figure 13, included SINS, single-axis turn-table, GPS, computers and a UPS. The SINS is manufactured by Harbin Engineering University, and the main parameters are shown in Tables 8 and 9. The single-axis turn-table is manufactured by Beijing Precision Engineering Institute for Aircraft Industry, and the main parameters are shown in Table 10.

### 5.1. Experimental Scheme

With the optimal design method proposed in this paper, the optimal angular rate for the experimental system is calculated to be 7 °/s. Therefore, three angular rates, 2 °/s, 7 °/s and 20 °/s, are used in the experiments for comparison. The experimental duration is 72 h, and the rotating scheme is the same as that in Section 4.1.

The rotating angular rate is not constant in a real system, since the IMU needs to reverse the rotating direction. The positioning accuracy of RSINS is influenced by the IMU turned angle in the variable angular rate process [26]. Additionally, the analysis results show that the error will be minimum in the process of the varied angle rate when the IMU's turned angle achieves 0.2*π.* In order to eliminate the effects of the variable angular rate, the angular accelerations are set as 0.1 °/s^2^, 0.7 °/s^2^ and 5.6 °/s^2^, respectively, in the experiment.

### 5.2. Experimental Results and Analysis

**Figure 14.**
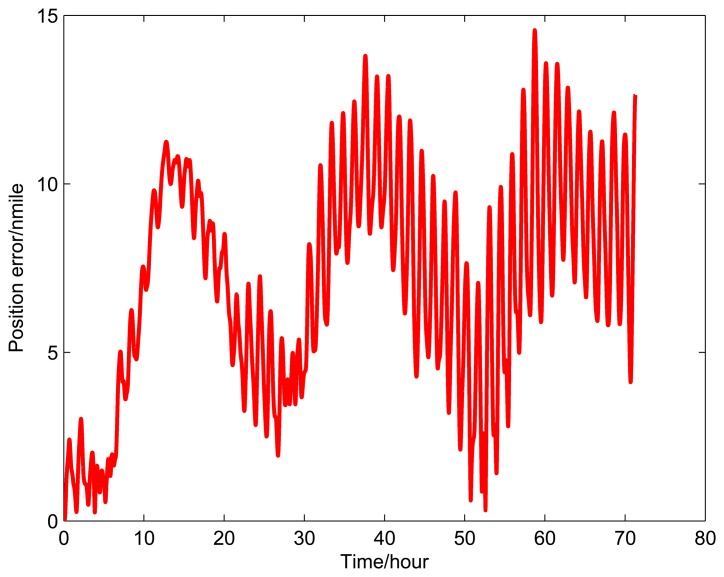
Experimental results (I).

**Figure 15.**
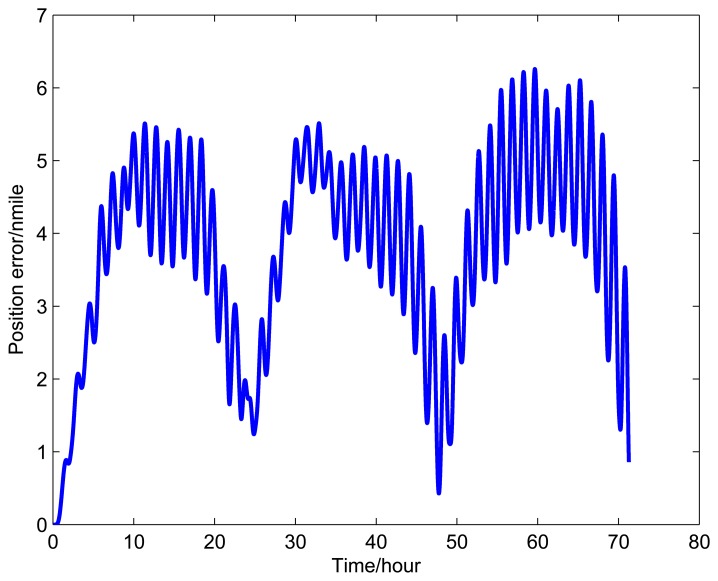
Experimental results (II).

**Figure 16.**
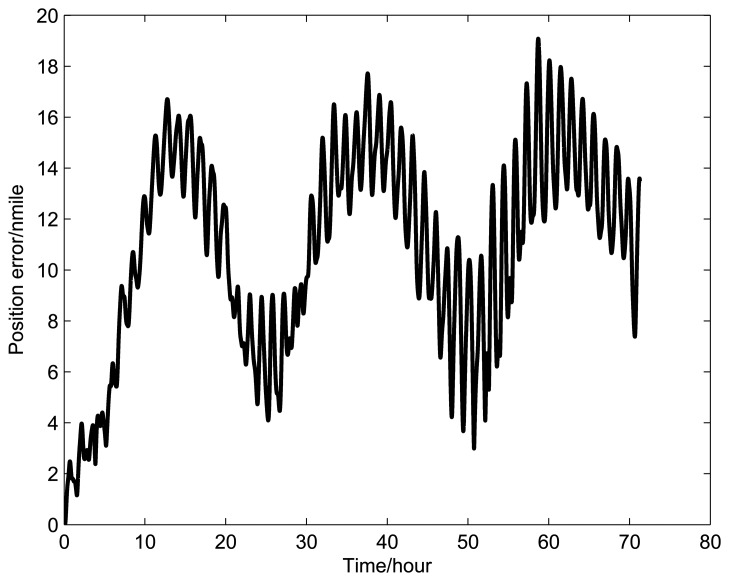
Experimental results (III).

Figures 14-16 show the position error of three experiments.

From the theoretical analysis of Section 3, it is known that the rotation modulation method can only reduce the amplitude of the oscillation error and the slope of the ramp error, but not eliminate them. Therefore, it can be seen from Figures 14-16 that the resulting position error comprises a ramp error with superimposed Schuler oscillation and Earth rotation oscillation, and the oscillation periods are 84.4 min and 24 h. From Table 11, it can be seen obviously that the position error of Experiment II is much less than the other two. The experimental results are consistent with the theoretical analysis of Section 3.

## 6. Conclusions and Future Work

This paper presented a rotating angular rate optimal design method of the RSINS. Firstly, the RSINS and the principle of the rotation modulation were introduced, and the inertial sensor errors were modeled. Secondly, the relation between the velocity error and the angular rate was worked out using the Laplace transform and the inverse Laplace transform. The optimal rotating angular rate method was also proposed based on the relationship above. Thirdly, simulations were conducted, and the results confirmed that the optimal rotating angular rate could be computed correctly with this method. Navigation experiments were done, and the results of the positioning error confirmed the effectiveness and correctness of the theoretical analyze. However, the experimental time was just 72 h, because of the limitations of experimental conditions. As a result, we plan to do more further experiments, which will last a longer time, if the experimental conditions permit, in the future. On the other hand, the results of the simulations were superior to that of the experiments. The reason may be that an error of the initial alignment, which is ignored, in the simulations may occur. The initial alignment error is not the main factor of the rotating angular rate optimal design method from the analysis of simulations and experimental results. Even so, we will model the initial alignment error and focus on its impact on the RSINS in the future.

## Figures and Tables

**Table 1.** Inertial sensor performance (Group 1).

**Sensor Error**	**Parameter Value**
Constant bias of gyroscope	0.01 (°/h)
Constant bias of accelerometer	9.8 × 10^-4^ (m/s^2^)
Scale factor error of gyroscope	1 × 10^-6^ (°/h)^-1^
Installation error of gyroscope	1 × 10^-9^ (°/h)

**Table 2.** Inertial sensor performance (Group 2).

**Sensor Error**	**Parameter Value**
Constant bias of gyroscope	0.05 (°/h)
Constant bias of accelerometer	4.9 × 10^-3^ (m/s^2^)
Scale factor error of gyroscope	0.2 × 10^-6^ (°/h)^-1^
Installation error of gyroscope	5 × 10^-9^ (°/h)

**Table 3.** Inertial sensor performance (Group 3).

**Sensor Error**	**Parameter Value**
Constant bias of gyroscope	0.002 (°/h)
Constant bias of accelerometer	1.96 × 10^-4^ (m/s^2^)
Scale factor error of gyroscope	5 × 10^-6^ (°/h)^-1^
Installation error of gyroscope	2 × 10^-10^ (°/h)

**Table 4.** Optimal rotating rates with different simulation conditions.

**Group**	**Optimal rotating angular rate**
1	21.6°/s
2	64.8°/s
3	7.2°/s

**Table 5.** Position error (Group 1).

Rotating angular rate (°/s)	7.2	21.6	45	64.8

Position error (nmile/72h)	1.2	0.6	1.5	1.3

**Table 6.** Position error (Group 2).

Rotating angular rate (°/s)	7.2	21.6	45	64.8

Position error (nmile/72h)	5.0	0.9	1.0	0.6

**Table 7.** Position error (Group 3).

Rotating angular rate (°/s)	7.2	21.6	45	64.8

Position error (nmile/72h)	2.5	3.0	5.0	4.2

**Table 8.** The main parameters of the fiber optics gyroscope.

**Performance**	**Parameters**
Dynamic range	±100°/s
Bias stability	≤ 0.003 °/h
Random walk	$\leq 0.001{}^{\circ}/\sqrt{h}$
Scale factor error	*≤* 5 ppm

**Table 9.** The main parameters of the accelerometer.

**Performance**	**Parameters**
Dynamic range	±4g
Bias stability	≤ 1 × 10^-5^

**Table 10.** Performances of the 920E single axis velocity and position turntable.

**Performance**	**Parameters**
Diameter	450 m
Carrying capacity	weight 50 kg
Rotating accuracy	±2″
Rotating range	continues and infinite
Location accuracy	±3″
Location definition	0.0001°
Speed range	0.005 - 200 °/s
Speed accuracy	5 × 10^-5^ (360°average)
Speed accuracy	5 × 10^-4^ (10°average)
Speed accuracy	1 × 10^-2^ (l°average)

**Table 11.** Experimental results.

Rotating angular rate (°/s)	2	7	20

Position error (nmile/72h)	14.7	6.4	19.6
